# Drug Repositioning Based on the Reversal of Gene Expression Signatures Identifies *TOP2A* as a Therapeutic Target for Rectal Cancer

**DOI:** 10.3390/cancers13215492

**Published:** 2021-10-31

**Authors:** Robson Francisco Carvalho, Luisa Matos do Canto, Sarah Santiloni Cury, Torben Frøstrup Hansen, Lars Henrik Jensen, Silvia Regina Rogatto

**Affiliations:** 1 Department of Clinical Genetics, University Hospital of Southern Denmark, 7100 Vejle, Denmark; luisa.matos.do.canto.alvim@rsyd.dk; 2 Institute of Regional Health Research, University of Southern Denmark, 5230 Odense, Denmark; 3 Department of Functional and Structural Biology—Institute of Bioscience, São Paulo State University (UNESP), Botucatu 18618-689, Brazil; santiloni.cury@unesp.br; 4 Department of Oncology, University Hospital of Southern Denmark, 7100 Vejle, Denmark; Torben.Hansen@rsyd.dk (T.F.H.); Lars.Henrik.Jensen@rsyd.dk (L.H.J.); 5 Danish Colorectal Cancer Center South, 7100 Vejle, Denmark

**Keywords:** rectal cancer, drug repositioning, reverse expression of signature genes, gene expression features, drug reversal potency scoring

## Abstract

**Simple Summary:**

Rectal cancer is the 8th most common cancer globally. Most patients with locally advanced rectal cancer receive neoadjuvant therapy based on 5-fluorouracil and radiotherapy showing variable responses. About 70–90% of patients present partial response, while 20% show treatment resistance. Repositioning drugs approved by regulatory agencies or drugs currently in clinical trials is a strategy to accelerate the development of drug-based cancer therapies. We compared rectal cancer gene expression signatures with reverse drug-induced gene-expression profiles of cancer cell lines to identify potential drugs for repositioning. Our analyses revealed that approved topoisomerase II inhibitors are candidate drugs for rectal cancer treatment. We also verified *TOP2A* copy number gains and increased expression in rectal tumors. These *TOP2A* alterations were independent predictive markers of topoisomerase inhibitor efficacy in colorectal cancer cells that closely represent rectal cancer signatures. Topoisomerase inhibitors are potentially helpful to treat rectal cancer patients with *TOP2A* imbalances.

**Abstract:**

Rectal cancer is a common disease with high mortality rates and limited therapeutic options. Here we combined the gene expression signatures of rectal cancer patients with the reverse drug-induced gene-expression profiles to identify drug repositioning candidates for cancer therapy. Among the predicted repurposable drugs, topoisomerase II inhibitors (doxorubicin, teniposide, idarubicin, mitoxantrone, and epirubicin) presented a high potential to reverse rectal cancer gene expression signatures. We showed that these drugs effectively reduced the growth of colorectal cancer cell lines closely representing rectal cancer signatures. We also found a clear correlation between topoisomerase 2A (*TOP2A)* gene copy number or expression levels with the sensitivity to topoisomerase II inhibitors. Furthermore, CRISPR-Cas9 and shRNA screenings confirmed that loss-of-function of the *TOP2A* has the highest efficacy in reducing cellular proliferation. Finally, we observed significant *TOP2A* copy number gains and increased expression in independent cohorts of rectal cancer patients. These findings can be translated into clinical practice to evaluate *TOP2A* status for targeted and personalized therapies based on topoisomerase II inhibitors in rectal cancer patients.

## 1. Introduction

Cancer is one of the leading causes of death worldwide [1]. Unfortunately, the discovery and translation of new drugs for cancer treatment is costly [2], requires years of research and development [3,4], and only a few drugs entering clinical trials are eventually approved for clinical use [5,6]. Drug repositioning has provided emerging opportunities for cancer therapeutic discoveries [7,8]. The definition of drug repositioning (also called drug repositioning, reprofiling, or re-tasking) has been explored since the publication of Ashburn and Thor in 2004 [9]. According to these authors, drug repositioning is the process of identifying and developing new uses for approved drugs or those under investigation outside the original indication [9]. The major advantages of repurposing old drugs are the use of de-risked compounds, with potentially shorter development time and reduced costs [8]. Therefore, repositioning drugs approved by regulatory agencies or currently in clinical trials are valuable alternatives for finding new cancer therapies that can be rapidly translated to the clinic [10]. This approach can benefit cancer patients by increasing the number of new therapeutic indications to non-oncology drugs or improving the effectiveness of personalized and targeted therapies [11].

Rectal cancer (ReCa) is the 8th most common cancer globally, with more than 300,000 deaths annually [1], and its incidence has risen exponentially in individuals aged between 18 and 50 years [12,13,14]. Most patients with locally advanced ReCa receive a complex multimodal therapy based on 5-fluorouracil and radiotherapy before surgery [15,16,17]. This treatment strategy was implemented in the mid-1990s [18] and has yielded an effective reduction in recurrence rates and improved survival [15]. Despite these improved outcomes, patients still suffer from high morbidity of the surgery [19,20], diverse side effects, and poor functional results of the treatment [21,22]. A complete pathological response is observed in only ~15% of patients after neoadjuvant chemoradiotherapy [23] a positive prognostic marker [24]. In the postoperative setting, medical treatment aims to decrease the risk of relapse and death [16,17]. However, only a minor fraction of the patients will benefit [25], culminating in the metastatic spread of ReCa, which is the leading cause of death [26]. More effective systemic treatments may increase the resectability of metastases and increase overall survival time. Thus, it is critical to identify new molecular markers to select patients for therapy and additional therapeutic strategies to treat ReCa.

Global gene-expression profiles are valuable resources for discovering potential therapeutic drug targets in human cancers [27,28]. Cancer gene-expression profiles are accumulating and available for exploration and reuse [29] through databases such as the Gene Expression Omnibus (GEO), The Cancer Genome Atlas (TCGA) [30], and the Cancer Cell Line Encyclopedia (CCLE) [31,32]. The integration of these cancer profiles with publicly available open data containing the effect of drugs on gene expression [28,33,34] or growth-inhibitory activity in human cancer cell lines [27] provides a valuable framework to computationally identify novel therapeutic candidates [33,35]. Different studies have combined drug-induced gene expression profiles with a cancer-specific expression profile to select compounds with high potency to reverse the expression of cancer-associated genes [36,37,38,39,40,41,42]. These drug-disease relationships found among anticorrelated expression profiles can benefit from using robust cancer gene expression signatures, which can be obtained by systematically analyzing cancer transcriptome datasets from several sources [43]. A data-mining of tumor gene-expression datasets allows the comparison of similar studies for consistency and identification of gene expression patterns [44], leading to biological discoveries and more accurate predictions of drug candidates for cancer treatment.

In this study, we first followed well-established criteria to select and reanalyze publicly available gene expression datasets of ReCa. Then, six signatures were obtained and used in a drug-repositioning bioinformatics approach to identify novel US Food and Drug Administration (FDA)–approved or investigational candidate drugs to treat ReCa. Using this strategy, we found that topoisomerase and cyclin-dependent kinase (CDK) inhibitors potentially reverse ReCa signatures and inhibit the growth of colorectal cancer (CRC) cell lines closely representing ReCa signatures. Thus, we demonstrate the reversal of ReCa gene expression as a strategy to rapidly identify drugs that are FDA-approved or currently in clinical trials to treat the disease.

## 2. Methods

### 2.1. Acquisition and Processing of Publicly Available ReCa Transcriptomic Datasets

We manually curated the GEO repository (https://www.ncbi.nlm.nih.gov/geo/) on 7 March 2021 to select the ReCa transcriptome datasets. The search strategy used the Medical Subject Headings (MeSH; https://www.ncbi.nlm.nih.gov/mesh/) (accessed on 2 March 2021) unique ID D012004 for Rectal Neoplasms (tumor or cancer of the rectum) from PubMed (Tables S1 and S2). We applied the following filters to select the datasets: Homo sapiens, gene expression platforms (Affymetrix, Agilent, and Illumina), with at least 30 rectal tissue samples (excluding formalin-fixed, paraffin-embedded tissues) from pre-treatment patients, with the surrounding normal tissues, studies indexed in PubMed and published in peer-reviewed journals from 2010 to 2021, and with standardized and annotated metadata. We also compared TCGA (https://www.cancer.gov/tcga) (accessed on 20 March 2021) RNA sequencing data from primary rectal adenocarcinoma (READ) [45] with RNA-Seq data from normal samples available in The Genotype-Tissue Expression (GTEx) program [46].

### 2.2. Generation of ReCa Signatures

We performed differential gene expression analyses between tumor and normal tissues for each cDNA microarray dataset using the interactive web tool GEO2R (https://www.ncbi.nlm.nih.gov/geo/geo2r/) (accessed on 10 March 2021), which is based on the *limma* package [47]. Next, we removed probes matching the same gene symbol by selecting the one with the lowest *p*-value. For the TCGA RNA-Seq dataset, the differential expression analysis between TCGA tumor and GTEx normal samples was performed using EdgeR [48] in the workspace Open Cancer TherApeutic Discovery (OCTAD) [49]. For this analysis, OCTAD uses a deep learning approach to select the top 50 highly correlated normal samples based on their gene expression profiles [50]. The final gene expression signature for each dataset was determined by applying the statistical cutoffs of Fold-change >1 and Adjusted *p*-value < 0.001.

### 2.3. Gene Set Enrichment Analysis

The final gene expression signature for each selected dataset and the list of differentially expressed genes (DEG) shared among all ReCa signatures were inputted into the EnrichR tool [51,52,53] for a comprehensive gene set enrichment analysis. Gene Ontology (Biological Process and Molecular Function), Kyoto Encyclopedia of Genes and Genomes (KEGG), NCI-Nature 2016 Pathway Interaction Database, ARCHS4 (Kinases Co-expression), and DisGeNET EnrichR libraries were used to identify genes compatible with drug targets potentially applicable to ReCa. The top five gene set enrichment terms for each library were included in consensus lists when satisfying the criteria of overrepresentation (Adjusted *p*-value < 0.05) in at least one signature. The enrichment results were represented by the *p*-value (Fisher’s exact test) and Z score (correction to the test) in a combined score computed by EnrichR [51,52]. To further explore commonly-enriched terms and their relationships, we used Metascape (https://metascape.org/) (accessed on 9 April 2021) [54] to perform a comparative analysis of all ReCa signatures. The enriched terms were represented by clusters with a similarity metric >0.3 determined by Kappa scores [55] and were displayed as nodes connected by edges in a network of overlapping enriched terms. Metascape selects the most statistically significant term within a cluster to represent the cluster. We also used Metascape [54] to identify protein-protein interaction (PPI) networks that were consistently altered in all ReCa signatures. Metascape uses PPI networks containing 3–500 proteins to detect densely connected network components using the Molecular Complex Detection (MCODE) algorithm [56].

### 2.4. Screening Drugs Targeting ReCa Using Gene Expression Signatures

The gene expression signature for each dataset was virtually screened for therapeutic targets using the OCTAD tool [49]. This platform matches cancer-specific expression signatures to compound-induced gene expression profiles (66,612 drug-induced gene expression profiles derived from 71 cell lines and 12,442 drugs) of the Library of Integrated Network-based Cellular Signatures (LINCS; L1000 dataset) [28,33,34]. The analysis is based on prioritizing small molecules with high potency to reverse the gene expression signature of the disease. OCTAD employs a summarization method to reduce bias and calculates the summarized Reversal Gene Expression Score (sRGES) [49]. The sRGES represents the reversal potency of a drug to a specific cancer signature. We selected the top 100 drug candidates based on the sRGES scores (lowest values < −0.25 in more than two drug-induced gene expression profiles) for the LINCS compounds (drugs in clinical trials or FDA-approved). These lists were compared with the results from the gene set enrichment analysis to generate a final list of drugs for further evaluation.

### 2.5. Genetic Dependencies of Target Drugs in CRC Cell Lines

To identify target genes for anticancer drugs and to discover the mechanistic basis of essentiality in specific cell types, we assessed the web tool shinyDepMap (https://labsyspharm.shinyapps.io/depmap) (accessed on 17 May 2021) [57]. This tool combines CRISPR and shRNA data available at the Cancer Dependency Map (DepMap; dataset version 19Q3) [58] to define, for each drug target gene, the growth reduction caused by knockdown/knockout (efficacy) and the selectivity of this effect across 423 cancer cell lines.

To systematically identify cancer vulnerabilities of selected drug targets, we further analyzed in vitro genetic dependencies in 53 CRC cell lines using DepMap CRISPR/Cas9 knockout screenings (DepMap 21Q2 Public + Score dataset) [59,60,61].

### 2.6. Drug Sensitivity in CRC Cell Lines

We also queried DepMap [58] to compare drug sensitivity between cancer cell lines to the selected drugs. We collected sensitivity data (PRISM Repurposing Primary and Secondary Screenings—19Q4) [27] for selected drugs showing high potency to reverse the ReCa-specific expression signatures in 35 CRC cell lines. PRISM is a 2-stage screening strategy that first screened 4518 drugs at a single dose (2.5 μM, in triplicate), and then 1448 drugs screened positive were re-screened in an 8-point dose-response (from 10 μM to 600 pM, in triplicate). Our drug sensitivity analysis based on these data prioritized drugs that effectively inhibited the growth of CRC cell lines with similar gene expression signatures to ReCa.

To measure this transcriptional similarity of the ReCa signatures and cancer cell lines to model anticancer drug sensitivity, we first used EnrichR to generate a consensus list based on the CCLE [31,32]. This CCLE consensus list includes the top 20 gene set enrichment terms (cell lines) from each signature that fulfilled the criteria of overrepresentation (Adjusted *p*-value < 0.05). We also compared the consensus list with tumor-cell line similarities estimated by Celligner (https://depmap.org/portal/celligner) (accessed on 6 May 2021) [62] and OCTAD [49]. Finally, the cancer cell lines were further classified into consensus molecular subtypes, according to Yu et al. 2019 [63].

### 2.7. Identification of Predictive Markers of Drug-Based Neoadjuvant Chemotherapy Efficacy

Copy number [log2 (relative to ploidy +1)] and RNA-Seq expression (TPM+1) data of CRC cell lines from the CCLE [31,32] were extracted from the DepMap [58] data portal (Copy Number 21Q2 Public and Expression 21Q2 Public datasets). Next, we calculated Pearson correlation coefficients for *TOP2A* and *TOP2B* copy number and gene expression with drug sensitivity data. This analysis was performed with topoisomerase inhibitors (doxorubicin, daunorubicin, idarubicin, epirubicin, teniposide, and mitoxantrone) in eight CRC cell lines, using the Correlation Matrix—online software: analysis and visualization (Statistical tools for high-throughput data analysis—STHDA; http://www.sthda.com/english/) (accessed on 19 May 2021).

Copy number alterations involving *TOP2A* were evaluated in 32 cases of locally advanced ReCa, as previously described [64]. Briefly, genomic profiling of the fresh-frozen pre-treatment biopsies was performed using the CytoScan HD array platform and analyzed with the Chromosome Analysis Suite (ChAS v.3.1, Affymetrix, Santa Clara, CA, USA). We considered significant alterations containing at least 25 probes altered for losses, 50 for gains, and a minimum of 5 Mb for cnLOH (copy-neutral loss of heterozygosity).

We analyzed the frequency of gene copy number and gene expression alterations of *TOP2A* in colorectal adenocarcinoma (TCGA, Firehose Legacy) patients using the cBioPortal (https://www.cbioportal.org/) (accessed on 20 May 2021) [65,66] database. Putative copy-number calls were determined using GISTIC 2.0 considered as −2: homozygous deletion, −1: hemizygous deletion, 0: neutral/no change, 1: gain, and 2: high-level amplification. Gene expression was represented by mRNA expression z-scores (RNA Seq V2 RSEM) compared to the expression distribution of each gene in tumors diploid for this gene. We included READ (*n* = 92), colon adenocarcinomas (COAD, *n* = 242), and mucinous adenocarcinomas of the colon and rectum (MUAD, *n* = 40).

### 2.8. Data Representation and Analysis

The intersection of DEG or drug lists among all signatures was analyzed using the Intervene Shiny App (https://intervene.shinyapps.io/intervene/) (accessed on 19 August 2021) [67]. This tool was also applied to determine the top DEG among the ReCa signatures based on the Manhattan distance. DEG shared by all signatures were displayed using a circos plot based on Metascape analysis [54]. The upstream regulatory network analysis of DEG shared among all signatures was generated with the eXpression2Kinases2 (X2K) Web (https://maayanlab.cloud/X2K/) (accessed on 14 April 2021) tool [68]. Heatmaps were created using the web tool Morpheus [69] (https://software.broadinstitute.org/morpheus). The ranking of the top 20 DEG and the volcano plots for each signature were generated in VolcanoseR (https://huygens.science.uva.nl/VolcaNoseR/) (accessed on 19 August 2021) [70].

## 3. Results

### 3.1. Acquisition and Processing of Publicly Available Transcriptomic Datasets for ReCa

**Figure 1:**
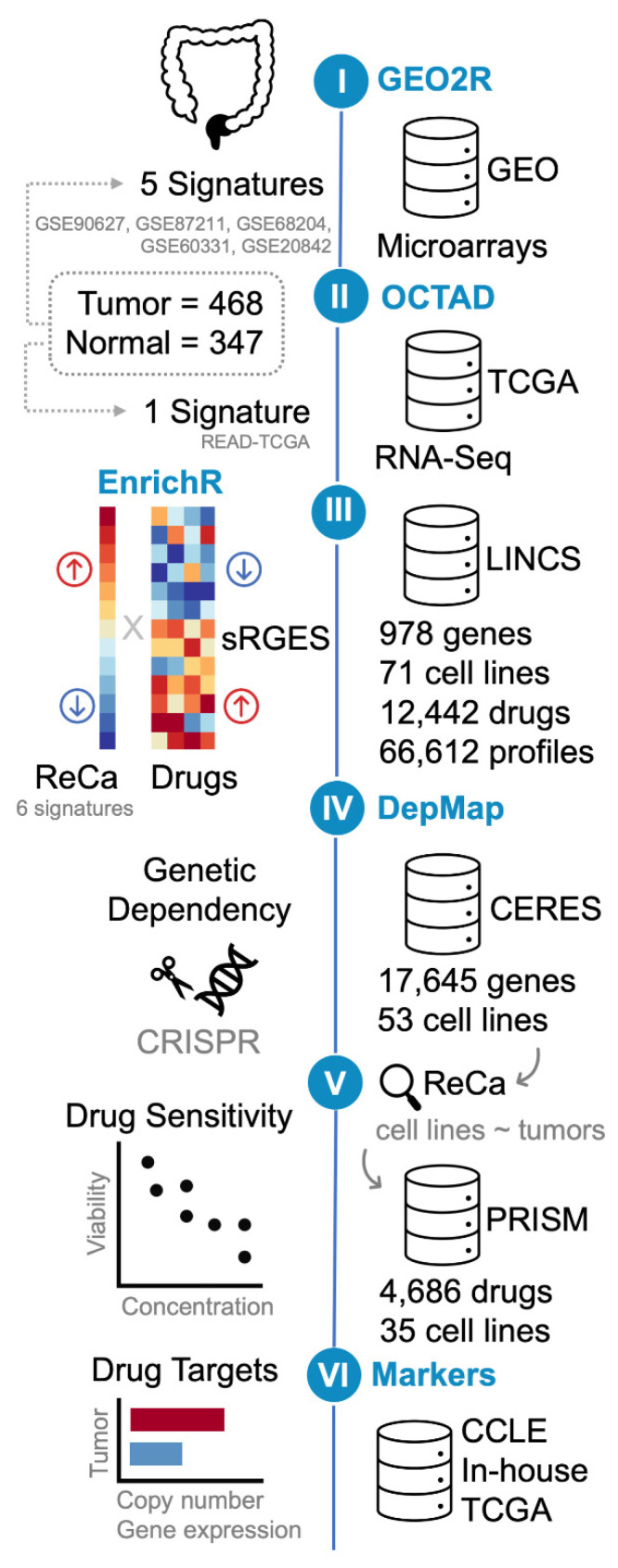
Schematic representation of the bioinformatics drug-repositioning approach (steps I-IV) using open data to identify drugs with potency to reverse gene expression signatures of rectal cancer (ReCa). The signatures were defined as the list of differentially expressed genes (DEG; log2-fold change > 1 and Adjusted *p*-value < 0.001) between tumor and normal samples. Step I: The reanalysis of microarrays transcriptome data available at Gene Expression Omnibus (GEO; GSE90627 [71], GSE87211 [72], GSE68204 [73], GSE60331 [74], and GSE20842 [75]) using GEO2R (https://www.ncbi.nlm.nih.gov/geo/geo2r/) (accessed on 10 March 2021) generated five signatures. Step II: The reanalysis of The Cancer Genome Atlas (TCGA) RNA-Seq data from primary rectal adenocarcinoma (READ) [45] resulted in one additional signature, which was created using the workspace “Open Cancer TherApeutic Discovery” (OCTAD; http://octad.org) (accessed on 5 May 2021) [49]. Step III: gene-set enrichment analysis was performed using EnrichR (https://maayanlab.cloud/Enrichr/) (accessed on 29 March 2021) [51,52,53]. OCTAD was also used to compare the six ReCa signatures to all the Library of Integrated Network-based Cellular Signatures (LINCS) drug-induced gene expression profiles [28,33,34]. OCTAD calculates the drug reversal potency based on a summarization method that quantifies a summarized Reversal Gene Expression Score (sRGES) to match anticorrelated signatures and drug-induced expression profiles. OCTAD was also used to generate target and chemical structure enrichment. Steps IV and V: The Cancer Dependency Map (DepMap; https://depmap.org/) (accessed on 11 June 2021) [27,58] was queried for discoveries of cancer cell lines vulnerabilities. The in vitro genetic dependencies of selected drug targets in cancer cell lines were analyzed using DepMap CRISPR/Cas9 knockout screenings (DepMap 21Q2 Public + Score, CERES dataset) [60,61] (Step IV). Colorectal cancer cell lines that present the most correlated transcriptomic profiles with primary ReCa samples, as determined by EnrichR [51,52,53] Cancer Cell Line Encyclopedia (CCLE) [31,32] library, Celligner [62], OCTAD [49], and Yu et al. [63] were selected for further analysis. The DepMap PRISM drug repurposing resource was used to validate the predictions using drug sensitivity data (PRISM Repurposing Primary and Secondary 19Q4 Screenings) [27] for the selected drugs showing high potency to reverse the ReCa-specific expression signatures in selected cancer cell lines (Step V). Step VI: Copy number and gene expression alterations of selected drug targets were assessed in colorectal cancer (CRC) cell lines (CCLE [31,32]) and tissues (TCGA [45] and in-house [64]) datasets to identify potential predictive markers of drug efficacy.

We explored and compared publicly available gene expression profiles of ReCa with those of multiple cancer cell lines treated with drugs in clinical trials or FDA-approved to identify drugs with potency to reverse gene expression signatures of the disease. The workflow of our analysis is summarized in Figure 1.

A total of six ReCa data sets comparing tumor (*n* = 468) and normal tissue (*n* = 347) samples was selected. Five of these ReCa datasets were generated using microarrays (GSE90627 [71], GSE87211 [72], GSE68204 [73], GSE60331 [74], and GSE20842 [75]) and one using RNA sequencing (RNA-Seq; TCGA;READ [45]) (Supplementary Data S1). The ReCa sample set comprises pre-treatment biopsy specimens, and the majority is represented by locally advanced ReCa. The description of each ReCa dataset and the corresponding clinical-pathological information is detailed in Tables S3 and S4, respectively.

Each selected microarray dataset was analyzed by comparing the ReCa samples with the respective normal tissues using the web tool GEO2R. For the TCGA RNA-Seq dataset, ReCa samples were compared with RNA-Seq data from normal samples available at GTEx Project [46]. The GTEx normal samples were selected using a deep learning approach based on gene expression profiles, available at the workspace OCTAD (http://octad.org) (accessed on 5 May 2021) [49]. The top 50 GTEx normal samples highly correlated with TCGA tumor samples [50] (Figure S1) were selected to perform differential gene expression analysis with OCTAD. The list of DEG (fold-change > 1 and adjusted *p*-value < 0.001) between ReCa and normal samples was defined as a ReCa signature for each dataset (Supplementary Data S2, Figure S2).

Among all microarrays-based signatures, the number of DEG in ReCa, relative to normal samples, varied from 2019 to 3326 genes (Figure S3a). As expected, RNA-Seq technology identified the highest number (4604) of DEG for the disease (Figure S3a).

### 3.2. Integrative Transcriptomic Analysis Reveals Cell Cycle Genes as Potential Drug Targets for ReCa

The essential idea of our bioinformatics strategy was to identify drugs that potentially reverse the ReCa signatures by decreasing genes that are overexpressed while stimulating those under-expressed. The key to such success is identifying specific targeted therapies, selecting drugs that influence the action or activity of a particular signaling pathway [76].

**Figure 2:**
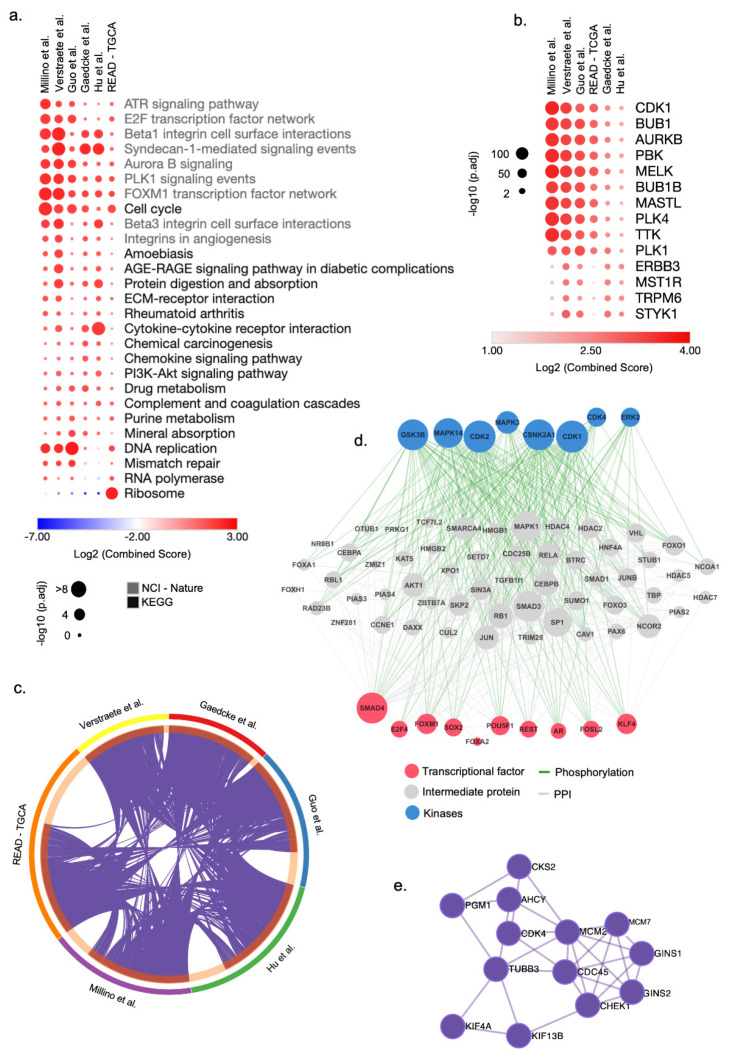
Integrative transcriptomic analysis reveals cell cycle genes as potential drug targets for rectal cancer (ReCa). (**a**) Top-ranked combined scores for The Kyoto Encyclopedia of Genes and Genomes (KEGG) and NCI-Nature 2016 pathway categories determined by the gene set enrichment analysis tool EnrichR [51,52]. The top five pathways for each category were included in a consensus list when satisfying the criteria of over-representation (Adjusted *p*-value < 0.05) in at least one signature. Columns and rows were clustered based on the Euclidean distance of log2 combined score values. (**b**) Top-ranked combined scores for kinase genes co-expressed with each ReCa signature in the ARCHS4 [78] database determined by the gene set enrichment analysis tool EnrichR [51,52]. The top five gene set enrichment terms were included in a consensus list when satisfying the criteria of over-representation (Adjusted *p*-value < 0.05) in at least one signature. Columns and rows were clustered based on Euclidean distance of log2 combined score values. (**c**) Circos plot visualization of the differentially expressed genes (DEG) that overlap among the ReCa signatures: Gaedcke et al., GSE20842 [75]; Guo et al., GSE90627 [71]; Hu et al., GSE87211 [72]; Millino et al., GSE68204 [73]; Rectal Adenocarcinoma from The Cancer Genome Atlas (TCGA) [45]; and Verstraete et al., GSE60331 [74]. The purple lines in the Circos plot connect DEG among multiple signatures. Each color in the outer circle represents one signature. The dark orange in the inner circle represents the number of overlapping DEG among the signatures. (**d**) The eXpression2Kinases2 (X2K) upstream regulatory network analysis [68] for a signature of 372 genes differentially expressed among all six ReCa signatures. The network includes transcription factors, proteins, and kinases predicted to regulate the expression of these 372 genes. (**e**) Protein-protein interaction network of the top enriched terms associated with DNA replication identified in all ReCa signatures by the Molecular Complex Detection (MCODE) algorithm in Metascape (https://metascape.org/) (accessed on 9 April 2021) [54].

To this aim, we first selected genes significantly deregulated in each signature to perform enrichment analysis using EnrichR. To facilitate a multi-platform analysis, we generated a consensus list comprising the top five pathways or terms for each category when satisfying the criteria of over-representation (Adjusted *p*-value < 0.05) in at least one signature. We observed a comprehensive spectrum of pathways and functional modules associated with cell cycle genes over-represented in all ReCa signatures (Figure 2a, Figures S4 and S5). The list of pathways includes the ATR signaling pathway, E2F transcription factor network, Beta1 integrin cell surface interactions, Syndecan-1-mediated signaling events, Aurora B signaling events, and DNA replication (Figure 2a). The gene ontology of the ReCa signatures also revealed the enrichment of biological processes and molecular functions associated with the cell cycle (Figure S6). The ARCHS4 kinase analysis showed an enrichment of several cell cycle kinases genes such as *CDK1, BUB1, AURKB, PBK, MELK, BUB1B, MASTL, PLK4, TKK,* and *PLK1* (Figure 2b), which constitute therapeutic targets for cancers [77]. *BUB1* and *MELK* were confirmed consistently deregulated in all ReCa signatures.

We found that most of the DEG are shared by at least two signatures (Figure 2c, Table S5), and 372 genes are deregulated in all six signatures (Figure S3a, Table S6). The upstream regulatory networks that regulate these 372 genes include transcription factors, proteins, and kinases associated with the cell cycle and DNA replication (Figure 2d,e). *CLDN1, CDH3, FOXQ1,* and *KRT80*, previously associated with CRC, were consistently placed in the top-ranked DEG among the signatures (Figures S2 and S3b). Interestingly, *CDK4* and the topoisomerases *TOP1MT* and *TOP2A* were included in the list of genes deregulated shared by all six signatures (Table S6). Additionally, *TOP1*, the target of the topoisomerase inhibitor irinotecan, was deregulated in four (Gaedcke et al. [75], Guo et al. [71], Hu et al. [72], and Verstraete et al. [74]) of six signatures (Table S5).

### 3.3. Topoisomerase and CDK Inhibitors Are Candidate Targets for Drug Repositioning in ReCa

We used a systems-based approach, available at the OCTAD [49], to compare ReCa signatures and drug-induced gene expression profiles from cancer cell lines (LINCS; L1000 dataset) to predict new therapeutic candidates. This analysis revealed the top compounds (in clinical trials or FDA-approved) that present high potency in reversing the expression of ReCa signatures (Supplementary Data S3, Figure S7).

**Figure 3:**
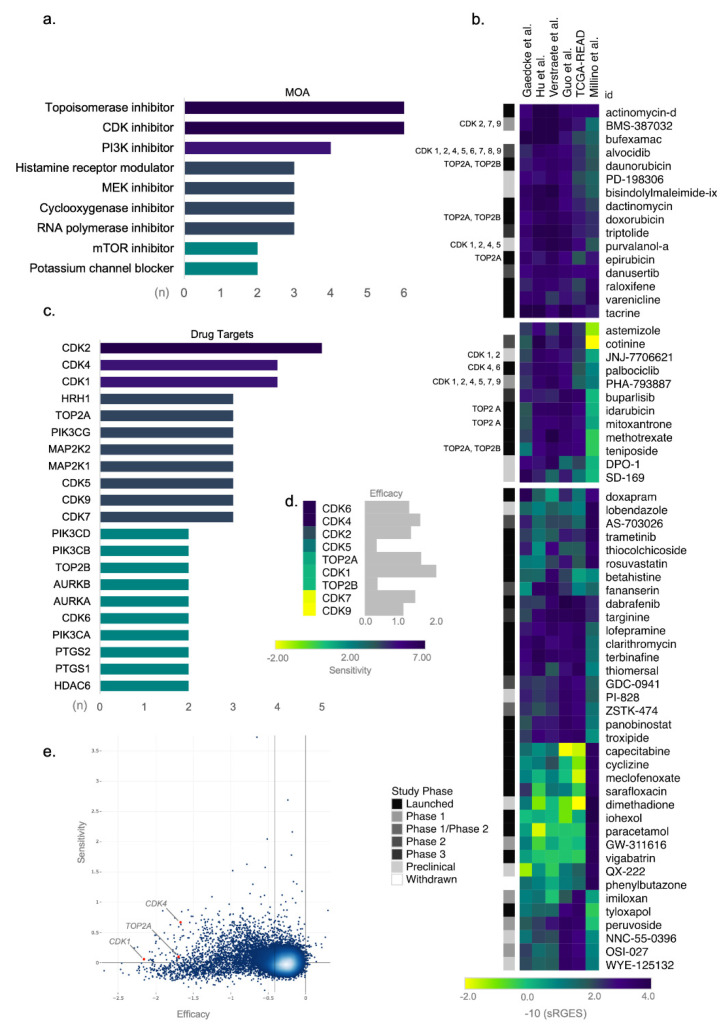
Topoisomerase and cyclin-dependent kinase (CDK) inhibitors as candidate targets for drug repositioning in rectal cancer (ReCa). (**a**) Drugs sharing similar mechanism of action (MOA) included among the top 100 compounds with the highest summarized Reversal Gene Expression Score (sRGES) determined by Open Cancer TherApeutic Discovery (OCTAD; http://octad.org) (accessed on 5 May 2021). (**b**) Heatmap of representative scores of overall reversal potency of a compound (sRGES) to ReCa signatures. The sRGES were clustered by the k-means clustering algorithm, and only the three clusters with the highest sRGES were displayed in the heatmap. The clinical phase of the drug development process for each compound is indicated in grayscale on the left side of the heatmap. Compounds targeting CDK and topoisomerase inhibitors are in the same two clusters with the highest sRGES. *CDK1, CDK2, CDK4, CDK5, CDK6, CDK7, CDK8,* and *CDK9* genes are indicated by 1, 2, 4, 5, 6, 7, 8, and 9, respectively. ReCa signatures are clustered by Euclidean distance between sRGES. (**c**) Drug targets for the top 100 compounds with the highest drug reversal potency score determined by OCTAD. We included only drug targets sharing the same specific target in the graph. (**d**) To predict the efficacy and selectivity of candidate drugs, the growth reduction caused by the loss of function of the *CDK1, CDK2, CDK4, CDK5, CDK6, CDK7, CDK8, CDK9, TOP2A*, and *TOP2B* genes (efficacy), and the selectivity of this effect across all cancer cell lines available at Cancer Dependency Map (DepMap) [58] were analyzed with the shinyDepMap tool (https://labsyspharm.shinyapps.io/depmap) (accessed on 17 May 2021) [57]. (**e**) Considering CDK and topoisomerase inhibitors, *CDK1* (1), *CDK4* (4), and *TOP2A* (A) showed high efficacy among 15.847 genes (blue dots). *CDK4* also presents high selectivity and constitutes a promising drug target for synthetic lethality of cancer cells.

To prioritize drugs predicted to reverse the ReCa gene expression signatures, we selected the top 100 compounds with the highest reversal potency score determined by OCTAD among all signatures (Table S7). Drugs that appeared in more than one signature were included once, resulting in a final list of 64 drugs (Table S8). Drugs targeting cell cycle pathways such as topoisomerase and CDK inhibitors had the highest number of drugs with a similar mechanism of action (six each) in our final list of top compounds (Figure 3a).

Topoisomerase and CKD inhibitors are also among the drugs with the highest drug reversal potency score (Figure 3b). The top-scored drugs predicted to reverse the ReCa gene expression are FDA-approved for cancer treatment, such as the topoisomerase inhibitors (daunorubicin, doxorubicin, epirubicin, idarubicin, mitoxantrone, and teniposide) and the CDK inhibitor (palbociclib) (Figure 3b). The list of these top-scored drugs also includes the CDK inhibitors currently in clinical trials (BMS-387032, alvocidib, purvalanol-a, JNJ-7706621, and PHA-793887) (Figure 3b). The six predicted topoisomerase inhibitors target *TOP2A* and *TOP2B*, whereas the *CDK2*, *CDK4*, and *CDK1* are the main targets of the six CDK inhibitors (Figure 3c).

Next, we used the shinyDepMap tool to evaluate the most promising topoisomerases and CDK genes as therapeutic targets by predicting their efficacy and selectivity in cancer cell lines. Among the CDK and topoisomerase inhibitors, those targeting *CDK1*, *CDK4*, or *TOP2A* showed the highest efficacies (Figure 3d). *CDK4* also presented high selectivity among 15.847 genes (Figure 3e), suggesting that this gene is a promising drug target for selective synthetic lethality of cancer cells. By explicitly analyzing CRISPR screening in 53 CRC cell lines, *TOP2A*, *CDK1*, *CDK7*, and *CDK9* presented the highest efficacies demonstrated by the most negative or toxic CERES effects (Figure S8).

We investigated similarities between gene expression profiles of ReCa and cancer cell lines. Using this in silico analysis, we tested the effects of selected drugs in cancer cell lines with the highest correlations to ReCa signatures. We first selected genes significantly deregulated in each signature to perform enrichment analyses using the CCLE [31,32] library in EnrichR [51,52,53] (Figure S9a). Our selection of representative cancer cell lines as models of rectal tumor signatures further used Celligner [62] data, which confirmed the alignment of the enriched CCLE CRC cell lines with ReCa tumor samples (Figure S9a,b). These similarity analyses allowed us to select a list of CRC cell lines with drug sensitivity data in the PRISM Repurposing Primary 19Q4 (SW1463, HT115, and CW2) or Primary and Secondary 19Q4 Screenings (HT55, SNUC4, LS80, LS1034, HCC56, SW498, CL34, and SNU61), available at DepMap (https://depmap.org/) [27,58] (Supplementary Data S4). The gene expression profiles for the cell lines SW948, CL34, HT115, and SNUC4 from the L1000 dataset were highly correlated with TCGA-READ gene expression profiles (Figure S9c), according to OCTAD results. The consensus molecular subtypes (CMS) classification, as described by Yu et al. [63], revealed cell lines matching the subtypes CMS1 (CW2), CMS2 (HCC56, HT55, LS1034, SW1463, SW948), and CMS3 (LC34, LS180, HT115, and SNU61) (Figure S9d). Moreover, the cell lines HCC56, HT55, and CL34 integrate the cancer cell line panel TCGA-110-CL, proposed by Yu et al. [63] for pan-cancer studies.

We used primary and secondary PRISM data available at DepMap [27,58] to verify the drug sensitivity of 11 cancer cell lines that presented similar transcriptional profiles to ReCa signatures. Fifty-eight of 64 drugs with the highest drug reversal potency score had available data in the PRISM (Supplementary Data S4). PRISM repurposing primary screening (single dose) had data for these 11 cell lines, and the secondary screening (8-point dose-response) had data for eight of them (Figures S10a,b, Supplementary Data S4). Notably, drugs that induced the highest sensitivity in these selected cancer cell lines in both primary and secondary screenings are inhibitors of topoisomerase (doxorubicin, idarubicin, epirubicin, daunorubicin, mitoxantrone, and teniposide) and CDK (BMS-387032, JNJ-7706621, alvocidib, PHA-793887, and palbociclib) (Figure S10). Among these drugs, idarubicin, BMS-387032, doxorubicin, epirubicin, teniposide, and mitoxantrone induced high sensitivity even at low doses (Figure S10).

### 3.4. TOP2A Gene Expression and Copy Number Gene Are Potential Predictive Markers of Topoisomerase Inhibitors Efficacy in ReCa

**Figure 4:**
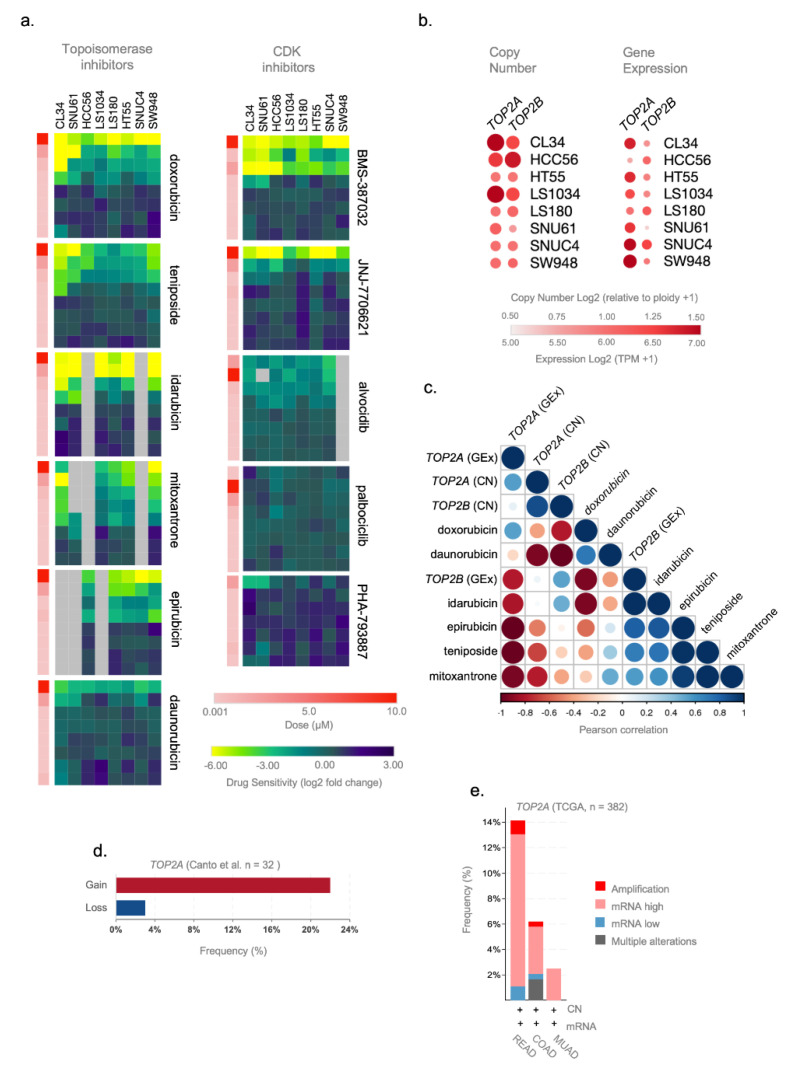
*TOP2A* gene expression and copy number are potential predictive markers of the efficacy of topoisomerase inhibitors in rectal cancer (ReCa). (**a**) Heatmaps of sensitivity (log2 fold change) for Topoisomerase and cyclin-dependent kinase (CDK) inhibitors in eight colorectal cancer (CRC) cell lines to validate the predictions using the PRISM Repurposing Secondary 19Q4 Screen [27,58]. The data were obtained in CRC cell lines with transcriptomic profiles similar to rectal tumor samples (Figure S8) and were downloaded from the Cancer Dependency DepMap; https://depmap.org/) (accessed on 11 June 2021) [58]. Columns (cells) and rows (drugs) for each compound were clustered based on the Euclidean distance of the log2 fold change values. The drug dose is represented in color bars and increases from light (0.001 µM) to dark red (10.0 µM). (**b**) Relative expression (TPM+1) and gene copy number [log2 (relative to ploidy +1)] of *TOP2A* and *TOP2B* in eight CRC cell lines from CCLE [31,32] database. The data (Copy Number 21Q2 Public and Expression 21Q2 Public datasets) were obtained in CRC cell lines with transcriptomic profiles highly correlated with rectal tumor samples (Figure S8) and downloaded from DepMap [58]. (**c**) Pearson correlation plot showing a negative correlation between *TOP2A* and *TOP2B* copy number and gene expression (Copy Number 21Q2 Public and Expression 21Q2 Public datasets) and drug sensitivity of topoisomerase inhibitors (doxorubicin, daunorubicin, idarubicin, epirubicin, teniposide, and mitoxantrone) (PRISM Repurposing Secondary 19Q4 Screen) in CRC cell lines (CL34, HCC56, HT55, LS1034, LS180, SNU61, SNUC4, and SW948). These colorectal cell lines present transcriptomic profiles highly correlated to rectal tumor samples (Figure S8). The molecular [31,32] and drug sensitivity [27] data were downloaded from DepMap [58]. (**d**) Frequency of copy number alterations (gain/loss) of *TOP2A* gene in a cohort of locally advanced rectal adenocarcinoma patients (*n* = 33) [64]. (**e**) Frequency of copy number and gene expression alterations of *TOP2A* gene in colorectal adenocarcinoma (TCGA, Firehose Legacy) patients using the cBioPortal (https://www.cbioportal.org/) (accessed on 20 May 2021) database. The alterations include amplification (red), mRNA high (pink), mRNA low (blue), and multiple alterations (gray). Putative copy number calls were determined using GISTIC 2.0 and considered the following values: −2 = homozygous deletion; −1 = hemizygous deletion; 0 = neutral/no change; 1 = gain; 2 = high level amplification. Gene expression is represented by mRNA expression z-scores (RNA Seq V2 RSEM) compared to the expression distribution of each gene tumor that is diploid for this gene. The analysis was performed using rectal adenocarcinoma (READ, *n* = 92), colon adenocarcinoma (COAD, *n* = 242), and mucinous adenocarcinomas of the colon and rectum (MUAD, *n* = 40).

We used the secondary PRISM Repurposing dataset containing cell line drug-perturbation viability screens for 11 topoisomerases and CDK inhibitors tested in eight CRC cell lines (CL34, SNU61, HCC56, LS1034, LS180, HT55, SNUC4, and SW948) that presented similar transcriptional profiles to ReCa signatures. Figure 4a depicts the treatment of these cell lines with eight concentrations of topoisomerases and CDK inhibitors (4-fold dilution starting from 10μM). By comparing the cellular vulnerabilities to topoisomerases and CDK inhibitors, we found that these cells were more sensitive to low doses of FDA-approved topoisomerase inhibitors (Figure 4a, Figure S10). Three cell lines (CL34, SNU61, and HCC56) showed the highest sensitivity and similar response to topoisomerase inhibitors (Figure 4a). Low doses of the CDK inhibitors BMS-387032 and JNJ-7706621 also induced high sensitivity in the eight selected CRC cell lines, but the FDA has not yet approved these two drugs. Based on the specific cellular vulnerabilities to FDA-approved topoisomerase inhibitors, we next focused on predictors of anticancer sensitivity for drugs targeting *TOP2A* and *TOP2B*. The genetic predictions of drug response can speed up the development of “personalized” therapeutic regimens [31]. Therefore, we examine *TOP2A* and *TOP2B* genes’ copy number and expression levels in CRC cell lines and tissues. Using CCLE data, we found gains of *TOP2A* and *TOP2B* in the cell lines CL34, HCC56, and LS1034; and increased *TOP2A* gene expression level in the cell lines CL34, SNU61, HCC56, LS1034, HT55, SNUC4, and SW948 (Figure 4b). We found clear inverse correlations between *TOP2A* gene copy number or expression levels with the sensitivity for topoisomerase inhibitors but not for *TOP2B*. Significantly, gains of *TOP2A* inversely correlated with the sensitivity of doxorubicin, daunorubicin, idarubicin, epirubicin, teniposide, and mitoxantrone (Figure 4c). Moreover, the expression levels of *TOP2A* inversely correlated with the sensitivity of daunorubicin, idarubicin, epirubicin, teniposide, and mitoxantrone (Figure 4c). Considering that some ReCa patients presented *TOP2A* gains (Figure 4d,e) or increased expression in ReCa compared to other colorectal tumors (Figure 4e), these events might constitute specific predictive markers of topoisomerase inhibitors efficacy in ReCa patients.

## 4. Discussion

The treatment option for most patients with locally advanced ReCa is neoadjuvant chemoradiation and surgery followed by adjuvant chemotherapy in selected cases [16,17]. However, only a few patients benefit from the treatment. Using a computational drug repositioning approach, we found that FDA-approved or investigational candidate drugs as new therapeutic options that may rapidly be translated to the clinics to treat ReCa. Among these drugs, topoisomerase (doxorubicin, idarubicin, epirubicin, daunorubicin, mitoxantrone, and teniposide) and CDK (BMS-387032, JNJ-7706621, alvocidib, PHA-793887, and palbociclib) inhibitors potentially reverse ReCa signatures and inhibit the growth of CRC cell lines with transcriptional profiles similar to ReCa. These cell lines presented higher sensitivity at low doses of topoisomerase inhibitors, which were correlated with the *TOP2A* gene expression and copy number. Therefore, we also propose *TOP2A* as a potential predictive marker of higher efficacy of topoisomerase inhibitors in ReCa patients. Together, our findings demonstrated that reversal gene expression analysis evinced drug targets and therapeutic alternatives for patients with ReCa. We consider that the computational workflow presented here can be applied with success to drug repositioning predictions in other cancer types.

Here, we present a global transcriptomic approach that used the OCTAD [49] tool to connect six ReCa signatures from GEO [71,72,73,74,75] and TCGA [45] datasets to 66,000 drug-induced gene expression profiles from the LINCS L1000 dataset [28,33,34]. Similar computational system biology approaches have been used to identify potential therapeutic targets for repositioning for hepatocellular carcinoma [38,42], Ewing sarcoma [39], basal cell carcinoma [40], renal cell cancer [41], small cell lung cancer, and neuroendocrine tumors [36]. Additionally, it has been demonstrated that the use of drug-induced gene expression profiles to determine the potency of a drug to reverse cancer gene expression signatures correlates with drug efficacy in preclinical models of breast, liver, and colon cancer [42]. The use of drug-induced gene expression profiles matched to inverse disease profiles has identified citalopram, an antidepressant, as a potential therapeutic option for patients with metastatic CRC [37]. Our approach generated a list of 64 FDA-approved or investigational candidate drugs that may potentially reverse the gene expression signature of ReCa. The topoisomerase and CKD inhibitors predominate among the FDA-approved drugs with the highest drug reversal potency score. Thus, the synergistic interaction of these drugs, when combined, should be better investigated.

To validate the effects of these 64 compounds in cancer cell lines as potential drugs to treat ReCa, we selected DepMap data from clinically relevant cancer cell lines, aiming to increase the likelihood of translating the preclinical findings. Recent studies have shown that cell lines differ in their ability to model primary tumors and that selecting cancer cell lines that recapitulate the pathobiology of tumors is critical [62,63]. Although some cancer cell lines are derived from ReCa, it is not possible to determine whether they were derived from patients undergoing multimodal therapy or from the actual anatomic rectum [79]. Therefore, we used cancer cell lines and tumor transcriptomics data [31,32,62,63] to select eight cell lines HT55, SNUC4, LS80, LS1034, HCC56, SW498, CL34, and SNU611 for dose-response screenings. These colorectal cell lines present gene expression profiles highly correlated with the gene expression signatures of ReCa samples.

Among our list of 64 compounds, inhibitors of topoisomerase (daunorubicin, doxorubicin, epirubicin, idarubicin, mitoxantrone, and teniposide) and CDK (palbociclib) are among the most potent drugs that induce cell sensitivity in selected cell lines. These results provide proof of the concept of our bioinformatics approach. Most notably, these drugs available for repurposing are FDA-approved or are in late-phase clinical trials for several malignancies. Although topoisomerases 2 and CDK4/6 inhibitors have emerged as a potent strategy for cancer treatment [80,81], few studies have tested these compounds in clinical trials for ReCa treatment. The CDK inhibitor palbociclib is under clinical trials phases 1 and 2 to treat CRC (ClinicalTrials.gov; NCT03981614, NCT01037790, NCT02465060, and NCT02897375). These studies warrant further attention considering that we found *CDK4* consistently deregulated across all ReCa signatures. The topoisomerase 2 inhibitor doxorubicin has also been tested in a terminated phase III clinical trial that evaluated the effectiveness of chemoembolization in treating patients with CRC metastatic to the liver (ClinicalTrials.gov; NCT00023868). Unfortunately, the results of this study were not published.

Topoisomerase 1 inhibitors are relevant drugs for ReCa treatment [80]. The combination of topoisomerase 1 inhibitor irinotecan with 5-fluorouracil has been proven effective for treating CRC [82]. Moreover, previous studies have demonstrated improved therapy efficacy combining irinotecan to the capecitabine or 5-fluorouracil chemoradiotherapy of locally advanced ReCa [83,84,85,86]. We found changes in the expression of the *TOP1* gene in four ReCa signatures, while *TOP1MT* and *TOP2A* were altered in all six signatures. Interestingly, two clinical trials (phases 1 and 2) that evaluated the use of topoisomerase 2 inhibitor epirubicin in CRCs highlighted that it induces better responses to rectal tumors [87,88].

The mechanisms involved in this specific response remain unclear and deserve further investigation. However, all cancer cell lines with transcriptional profiles comparable to ReCa signatures presented high sensitivity to topoisomerase inhibitors (idarubicin, doxorubicin, epirubicin, teniposide, and mitoxantrone), even at low doses. Based on the high sensitivity of the cell lines tested to topoisomerase inhibitors and that these drugs are all approved by the FDA, rectal cancer patients may also be benefited from this treatment. Moreover, the dysregulation of *TOP2A* found in all ReCa signatures suggests its role as a marker of anticancer sensitivity for these drugs. *TOP2A* amplification is a known predictive marker of anthracycline-based neoadjuvant chemotherapy efficacy in patients with breast cancer [89,90]. We found correlations between *TOP2A* gene imbalances and expression levels and the sensitivity of CRC cell lines for topoisomerase inhibitors. We also confirmed that *TOP2A* gene amplification and increased expression are frequently detected in ReCa samples [91,92,93,94]. Therefore, testing the *TOP2A* status may predict more accurately the patients that likely benefit from treatments based on topoisomerase inhibitors. Accordingly, the clinical benefit of epirubicin treatment was recently tested in patients with metastatic CRC resistant to oxaliplatin and *TOP2A* amplification [95]. However, only six patients were enrolled in the study, and the results were not conclusive. Based on our data, we propose that topoisomerase inhibitors identified here are promising candidates for repositioning to advance the ReCa treatment. These drugs may specifically benefit ReCa patients with amplification and increased expression of the *TOP2A* gene.

Besides predicting drug candidates, our comprehensive and integrated characterization of the six ReCa signatures revealed marked similarities in gene expression and pathways deregulated in the disease. Pathways associated with cell cycle and DNA replication genes were over-represented in all ReCa signatures. The list of oncogenic pathways that can be targeted to treat ReCa includes the ATR signaling pathway [96], E2F transcription factor network [97], Beta1 integrin cell surface interactions [98], Syndecan-1-mediated signaling events [99], Aurora B signaling events [100,101], and DNA replication [102,103]. We identified genes consistently altered in all ReCa signatures, such as the top-ranked DEG *CLDN1, CDH3, FOXQ1,* and *KRT80*, all previously associated with CRC [104,105,106,107]. The consistent deregulation of these pathways and genes such as *CDK4* and *TOP2A* reinforces their relevance as potential therapeutic targets for ReCa.

In conclusion, we showed that mining and integrating publicly available open data of cancer gene expression profiles with the effect of drugs on gene expression or inhibitory activity in human cancer cell lines is a promising strategy for the computational repositioning of compounds to ReCa treatment. Based on the generated data, we propose that topoisomerase inhibitors (such as idarubicin, doxorubicin, epirubicin, teniposide, and mitoxantrone) should be tested in a specific cohort of ReCa patients with increased expression or amplification of the *TOP2A* gene. Alternatively, these drugs or their combinations with the top hits here identified can be screened in rectal cancer-derived organoids [108]. Pre-clinical studies using cancer-derived organoids to drug testing are valuable in predicting drug responses in a clinical setting. These in vitro models are known to more accurately recapitulate the tumoral biological heterogeneity associated with patient-specific cancers [109], allowing a diversity of drug-screening applications [110].

## Data Availability

The datasets reanalyzed during the current study are available in the GEO (https://www.ncbi.nlm.nih.gov/geo/; GSE90627, GSE8721145, GSE6820446, GSE6033147, and GSE2084248), TCGA (https://portal.gdc.cancer.gov/; TCGA-READ), LINCS (https://lincsproject.org/), and DepMap (https://depmap.org/) repositories. The data generated during this study are included in this published article and its supplementary information files.

## Supplementary Materials

The following are available online at https://www.mdpi.com/article/10.3390/cancers13215492/s1: Supplementary Data S1: Microarrays (GEO) and RNA-Seq (TCGA) data of rectal cancer datasets included in this study, Supplementary Data S2: List of differentially expressed genes (fold-change > 1 and adjusted *p*-value < 0.001) between rectal cancer and normal samples, Supplementary Data S3: OCTAD output files with the sRGES (summarized Reversal Gene Expression Score) for rectal cancer gene expression signatures, Supplementary Data S4: Sensitivity data (PRISM Repurposing Primary and Secondary Screenings—19Q4) for selected drugs showing high potency to reverse the rectal cancer-specific expression signatures in 35 colorectal cancer cell lines, Table S1: Medical Subject Headings (MeSH) unique ID D012004 for Rectal Neoplasms (tumors or cancer of the rectum) retrieved from PubMed (https://www.ncbi.nlm.nih.gov/mesh) (accessed on 2 March 2021), Table S2: Medical Subject Headings (MeSH) unique IDs and GEO datasets Study IDs for rectal cancer, Table S3: Description of the rectal cancer GEO datasets included in this study, Table S4: Clinical and pathological data of rectal cancer patients investigated in this study. Table S5: Differentially expressed genes (fold-change > 1 and adjusted *p*-value < 0.001) shared in rectal cancer signatures, Table S6: Differentially expressed genes (fold-change > 1 and adjusted *p*-value < 0.001) shared in six rectal cancer signatures, Table S7: Top 100 compounds with the highest reversal potency score (sRGES < −0.25; *n* > 2) determined by OCTAD among the six rectal cancer signatures, Table S8: Top 64 compounds (unique, from the top 100) with the highest reversal potency score determined by OCTAD among the six rectal cancer signatures, Figure S1: Selection of reference normal samples from The Genotype-Tissue Expression (GTEx) to generate rectal cancer (ReCa) gene expression signature for primary rectal adenocarcinoma (READ) samples from The Cancer Genome Atlas (TCGA) [45]. The GTEx normal samples that highly correlated with READ samples were selected in the workspace “Open Cancer TherApeutic Discovery” (OCTAD; http://octad.org) (accessed on 5 May 2021) [49]. This OCTAD analysis selected the top 50 normal samples (red dots) using the Spearman correlation test of top varying genes between each READ-TCGA sample and all GTEx normal samples (gray dots), Figure S2: Rectal cancer (ReCa)-specific gene expression signatures, Volcano plots showing fold changes (Log2 FC > 1) for differentially expressed genes (DEG; FDR-adjusted *p*-value < 0.01) between ReCa and normal samples. The volcano plots summarize results for Gaedcke et al. (GSE20842) [75], Millino et al. (GSE68204) [73], Verstraete et al. (GSE60331) [74], Hu et al. (GSE87211) [72], Guo et al. (GSE90627) [71], and TCGA-READ [45]. The top 20 ranking genes indicated in the volcano plots for each signature were calculated using the Manhattan distance. *CLDN1, CDH3, FOXQ1,* and *KRT80*, previously associated with CRCs, were consistently placed in the top-ranked DEG (green dots), Figure S3: Differentially expressed genes shared among all rectal cancer (ReCa) signatures identified as top-deregulated genes previously described in colorectal cancer (CRC). (a) Upset plot showing the number of differentially expressed genes (DEG; red bars) shared by all ReCa signatures. The blue bar graphs depict the number of DEG in each signature. The combinations of shared DEG among bar plots are indicated in the grid (bottom). (b) Upset plot showing the intersections of the top 20 DEG (red bars) among ReCa signatures determined based on the Manhattan distance. *CLDN1, CDH3, FOXQ1,* and *KRT80* genes (bold), previously associated with CRCs, were consistently placed in the top-ranked DEG among the signatures, Figure S4: Network of overlapping enriched terms among rectal cancer (ReCa) signatures, performed by Metascape (https://metascape.org/) (accessed on 9 April 2021) [54]. (a) The enriched terms are colored according to the ID of each cluster. The clusters consist of enriched terms with a similarity metric > 0.3 determined by Kappa scores and are connected by edges. The nodes sharing the same cluster ID are generally next to each other. (b) The enriched terms are colored according to *p*-values. Terms containing more genes tend to have a more significant *p*-value, Figure S5: Protein-protein interaction network analysis based on overlapping significant differentially expressed genes (DEG) in all rectal cancer (ReCa) signatures, performed by Metascape (https://metascape.org/) (accessed on 9 April 2021) [54]. Networks containing between 3 and 500 proteins were used to detect densely connected network components using Molecular Complex Detection (MCODE) algorithm, Figure S6: Top-ranked combined scores for gene ontologies (GO) terms determined by the gene set enrichment analysis tool EnrichR [51,52,53]. The top five terms for each biological process (BP) and molecular function (MF) were included in a consensus list according to the adopted criteria of over-representation (Adjusted *p*-value < 0.05) in at least one signature. Columns and rows were clustered based on Euclidean distance based on log2 combined score values, Supplementary Figure S7: Identification of top compounds in clinical trials or FDA-approved that present high potency to reverse the expression of six rectal cancer (ReCa) signatures. The first column of each heatmap shows a ReCa signature: Gaedcke et al., GSE20842 [75]; Millino et al., GSE68204 [73]; Verstraete et al., GSE60331 [74]; Hu et al., GSE87211 [72]; Guo et al., GSE90627 [71]; and Rectal Adenocarcinoma (READ) from The Cancer Genome Atlas (TCGA) [45]. In this first column, genes that are upregulated and downregulated in ReCa, compared to normal samples, are shown in red and blue, respectively. The remaining columns of each heatmap show the top LINCS L1000 drug signatures [28,33,34] with a reverse expression profile of the corresponding ReCa signature (first column). In these drug signature columns, red and blue indicate genes with high and low expression induced by drug treatment, respectively, Figure S8: CERES [60] effects for CRISPR knockout screening of *CDK1, CDK2, CDK4, CDK5, CDK6, CDK7, CDK8, CDK9, TOP2A*, and *TOP2B* genes among 53 colorectal cancer (CRC) cell lines available at Cancer Dependency Map (DepMap; 21Q2 Public + Score dataset) [58]. Columns and rows were clustered using Euclidean distance based on the gene effect (CERES) values, Figure S9: Selection of representative cancer cell lines as models for rectal tumors. (a) Top-ranked combined scores for colorectal cancer (CRC) cell lines from Cancer Cell Line Encyclopedia (CCLE) [31,32] presenting the most correlated transcriptomic profiles with primary rectal cancer (ReCa) samples. This gene set enrichment analysis was performed using EnrichR (https://maayanlab.cloud/Enrichr/) (accessed on 13 April 2021) [51,52,53]. Cell lines with drug sensitivity data in the PRISM Repurposing Primary 19Q4 (gray arrowhead) or Primary and Secondary 19Q4 Screenings (black arrowhead) available at the Cancer Dependency Map (DepMap; https://depmap.org/) (accessed on 11 June 2021) [27,58] were selected for further analyses. The cell lines that closely resembled rectum adenocarcinomas (shown in b—connecting lines) were queried to discover cancer cell line vulnerabilities for selected drugs showing high potency to reverse the ReCa-specific expression signatures. (b) ReCa cell lines from CCLE [31,32] that closely resemble rectal adenocarcinomas, as determined by Celligner [62] (https://depmap.org/portal/celligner/) (accessed on 6 May 2021). This method applies an unsupervised approach that corrects differences when integrating large-scale cell line and tumor RNA-Seq datasets. The distance between a cell line and rectal adenocarcinomas, based on median Euclidean distances, decreases from blue to red according to the color bars. The cell lines with drug sensitivity data in the PRISM Repurposing Primary 19Q4 (gray arrowhead) or Primary and Secondary 19Q4 Screenings (black arrowhead) available at the DepMap [27,58] were selected for further analyses. (c) Spearman rank correlation between gene expression profiles from the Library of Integrated Network-based Cellular Signatures (LINCS) L1000 data set [28,33,34] and The Cancer Genome Atlas (TCGA) RNA-Seq data for primary rectal adenocarcinoma (READ) [45] samples was estimated by the workspace “Open Cancer TherApeutic Discovery” (OCTAD; http://octad.org) (accessed on 5 May 2021) [49]. This OCTAD analysis is based on the average of correlations between the LINCS cell lines and individual tumors. This analysis also revealed cell lines with drug sensitivity data in the PRISM Repurposing Primary 19Q4 (gray arrowhead) or Primary 19Q4 and Secondary 19Q4 Screenings (black arrowhead), available at the DepMap [27,58]. (d) Prediction of cancer cell line subtypes using gene expression-based consensus molecular subtypes (CMS) of colorectal adenocarcinoma tumors. The data obtained from Yu et al. [63] is based on correlations between transcriptomic profiles from TCGA [45] and the CCLE [31,32]. Selected cell lines (in bold) with drug sensitivity data in the PRISM Repurposing Primary 19Q4 or Primary 19Q4 and Secondary 19Q4 Screenings available at the DepMap [27,58] are indicated as gray or black arrowheads, respectively. The cell lines HCC56, HT55, and CL34 (indicated as asterisks) are included in the cancer cell line panel TCGA-110-C [63], Figure S10: Drug sensitivity for the 58 selected drugs showing high potency to reverse rectal cancer (ReCa)-specific expression signatures. The data were obtained from colorectal cancer (CRC) cell lines with transcriptomic profiles highly correlated with rectal tumor samples (Figure S9) (downloaded from the Cancer Dependency Map, DepMap; https://depmap.org/) (accessed on 11 June 2021) [27,58]. (a) Heatmap of drug sensitivity (log2 fold change) in 11 CRC cell lines to validate the predictions using the PRISM Repurposing Primary 19Q4 Screen [27,58]. (b) Heatmaps of drug sensitivity (log2 fold change) in eight CRC cell lines to validate the predictions using the PRISM Repurposing Secondary 19Q4 Screen [27,58]. Columns (cells) and rows (drugs) were clustered based on the Euclidean distance of the log2 fold change values. The complete heatmap on the right has 280 rows intervals; however, only the first top 74 (selection indicated by red dashed line), with the corresponding drug dose, are shown on the left for visualization purposes. According to the color bars, drug dose increases from light (0.001 µM) to dark red (10.0 µM).
