# Supplementary figures and images for: Drug Repositioning Based on the Reversal of Gene Expression Signatures Identifies TOP2A as a Therapeutic Target for Rectal Cancer
Source: Cancers (Basel). 2021 Oct 31;13(21):5492. doi: 10.3390/cancers13215492 (PMC8583090; doi:10.3390/cancers13215492)

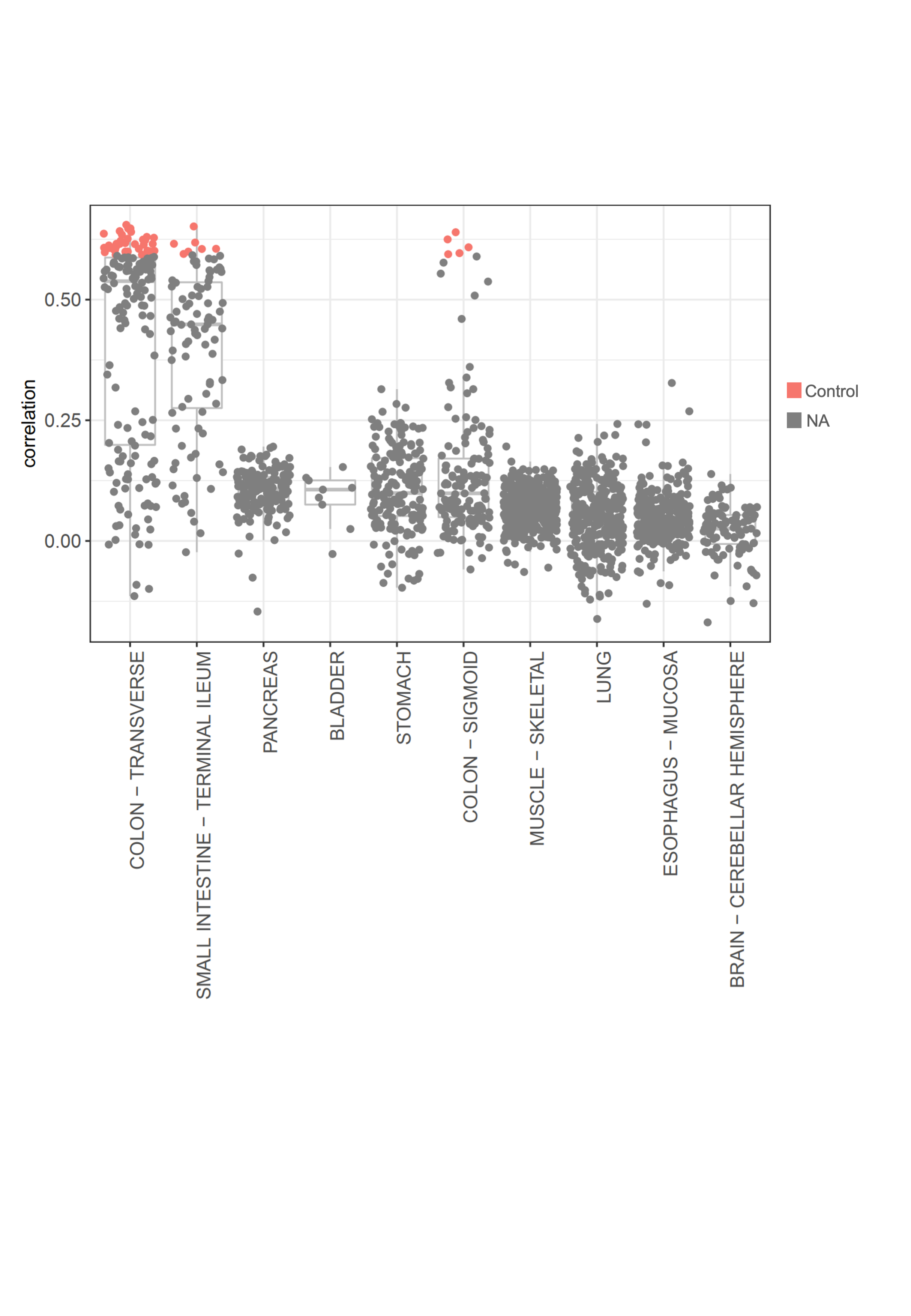

Supplement: Supplementary file 1 [file cancers-13-05492-s001.zip › Supplementary Figure S1.tiff]

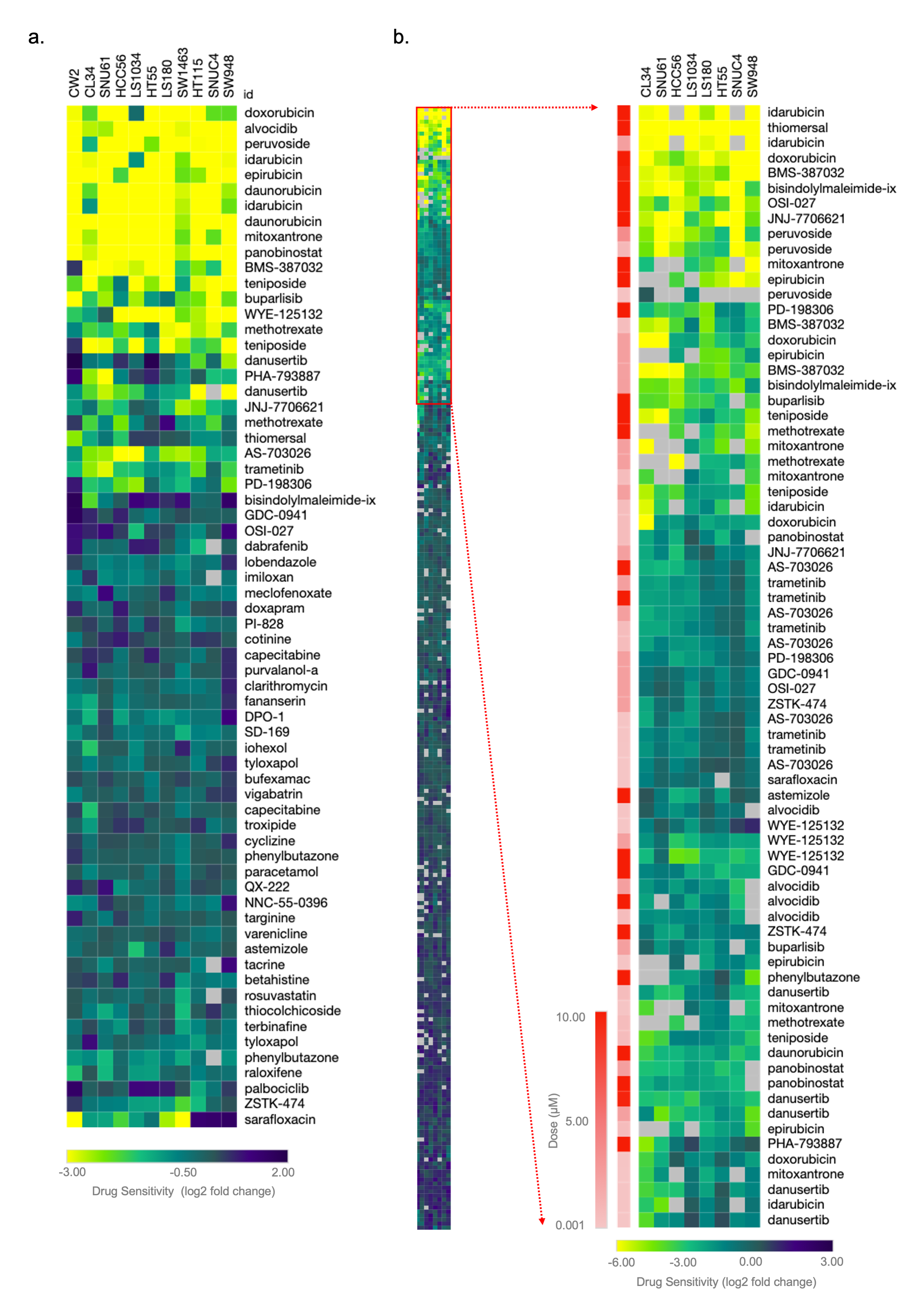

Supplement: Supplementary file 1 [file cancers-13-05492-s001.zip › Supplementary Figure S10.tiff]

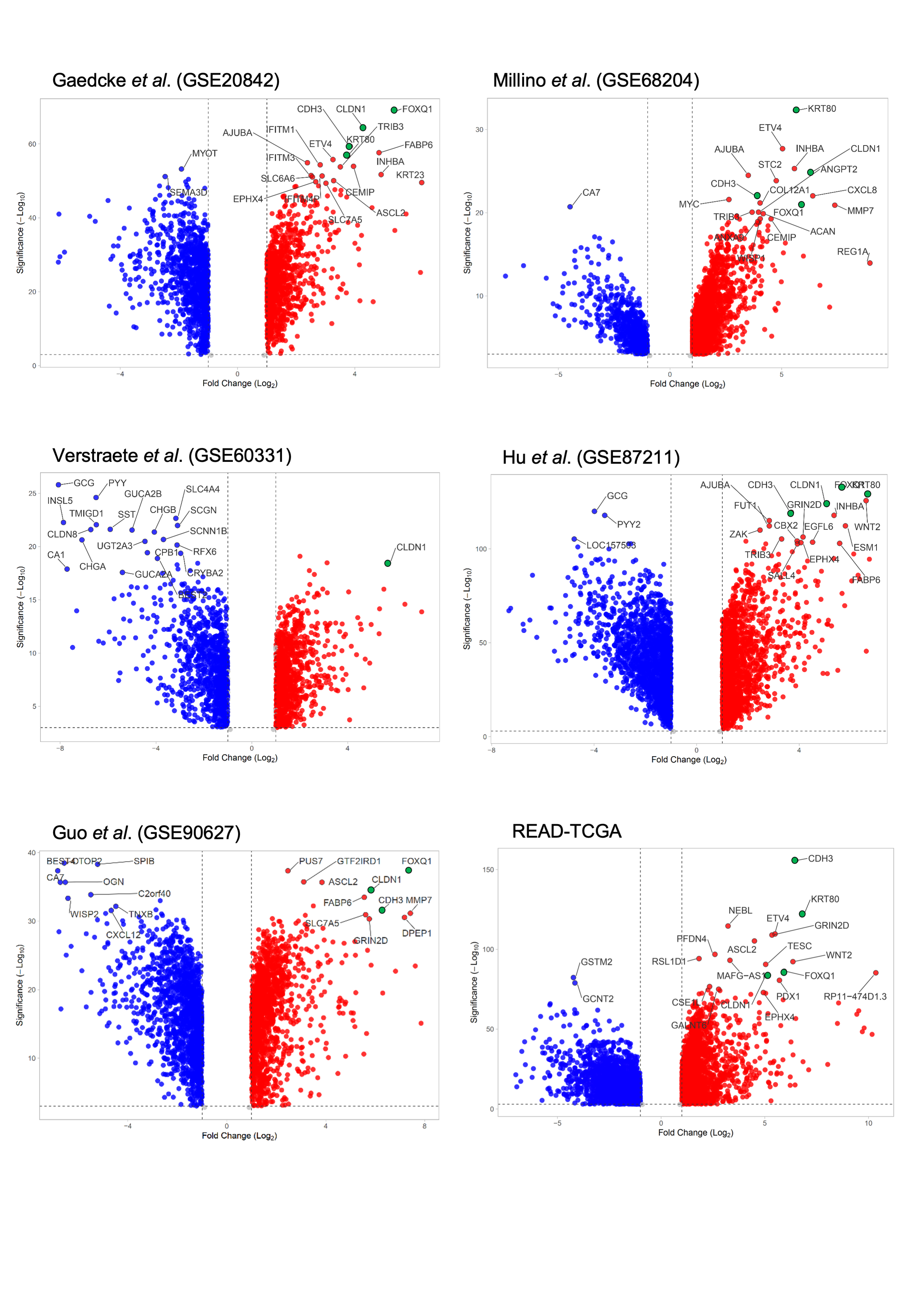

Supplement: Supplementary file 1 [file cancers-13-05492-s001.zip › Supplementary Figure S2.tiff]

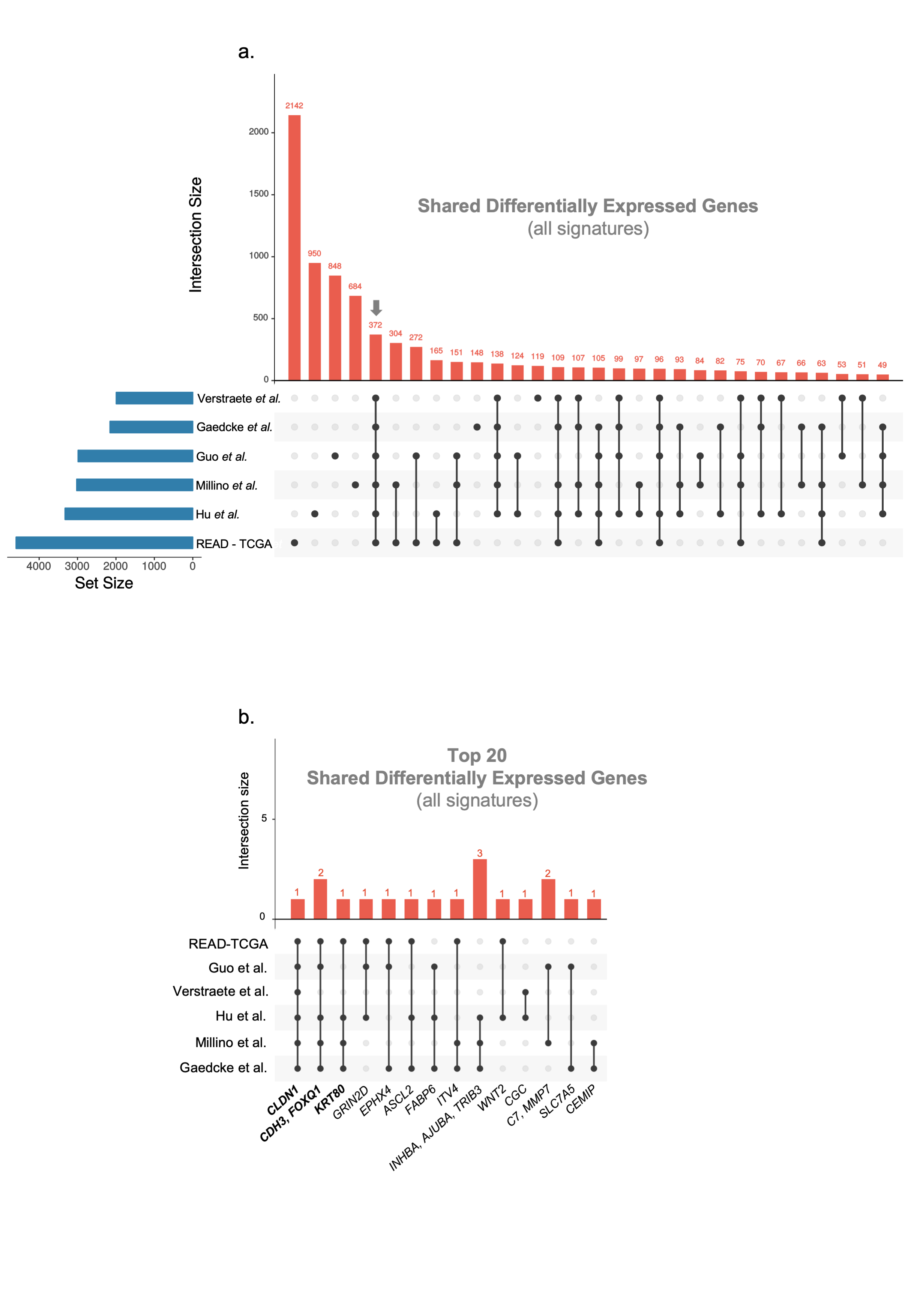

Supplement: Supplementary file 1 [file cancers-13-05492-s001.zip › Supplementary Figure S3.tiff]

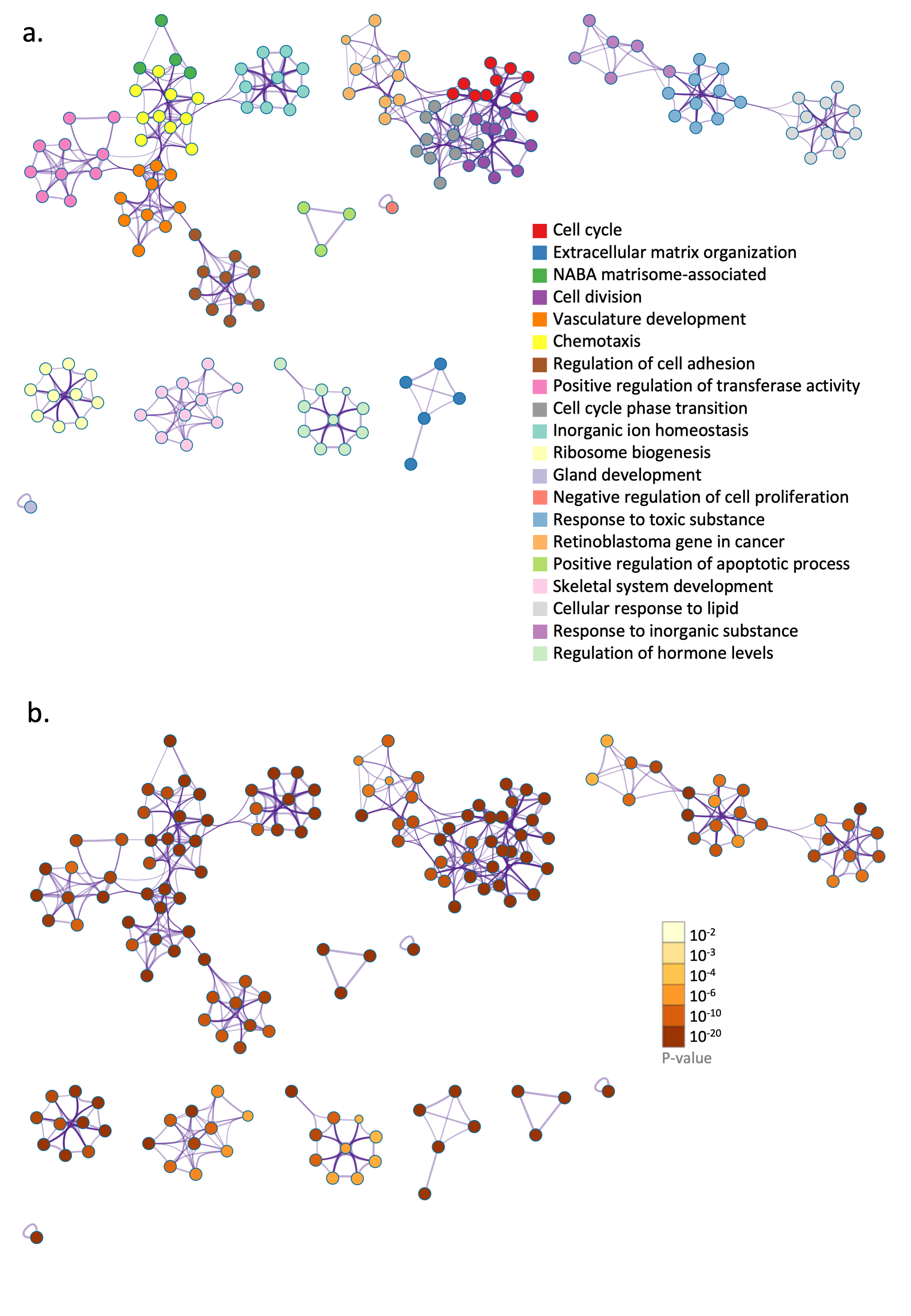

Supplement: Supplementary file 1 [file cancers-13-05492-s001.zip › Supplementary Figure S4.tiff]

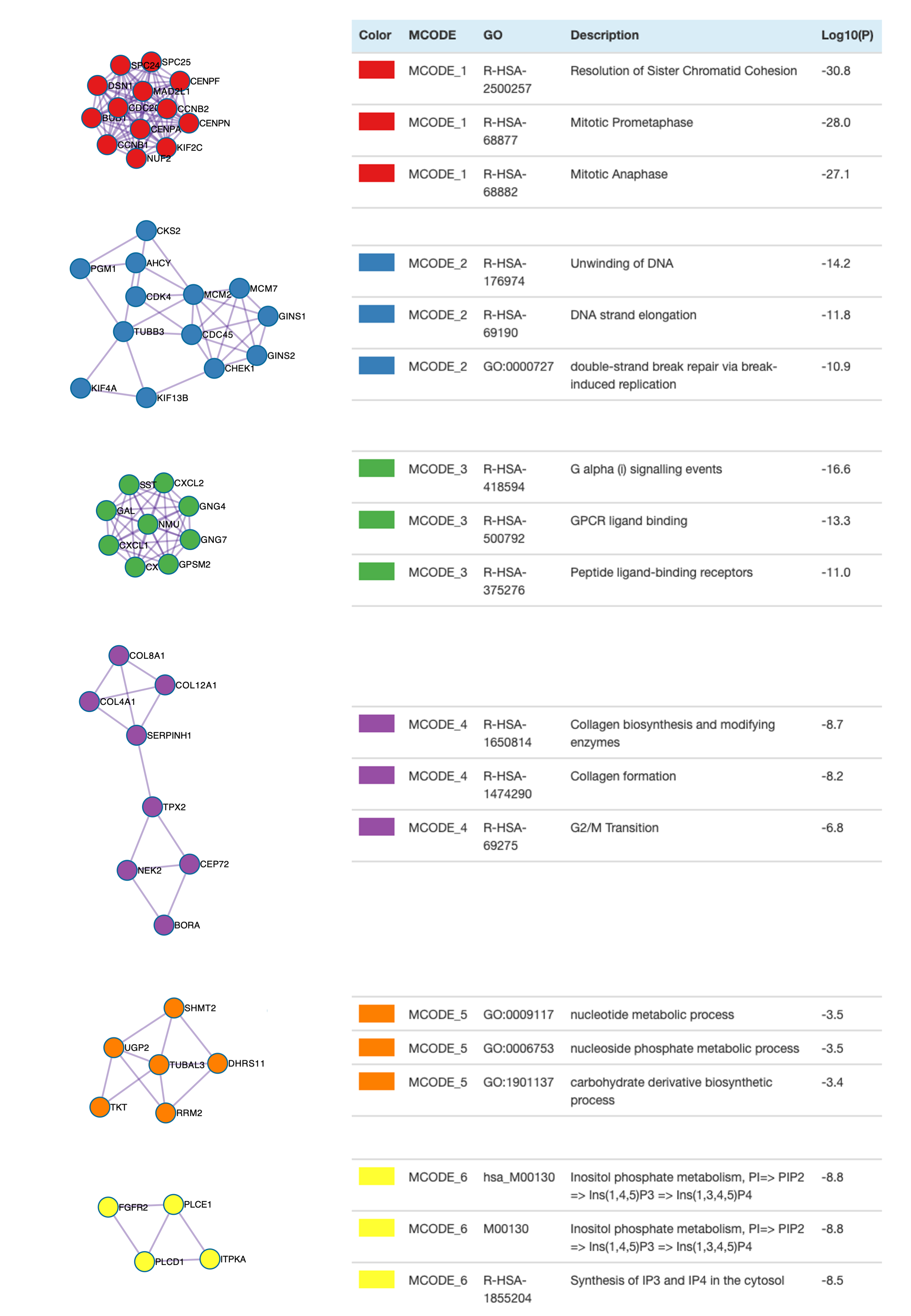

Supplement: Supplementary file 1 [file cancers-13-05492-s001.zip › Supplementary Figure S5.tiff]

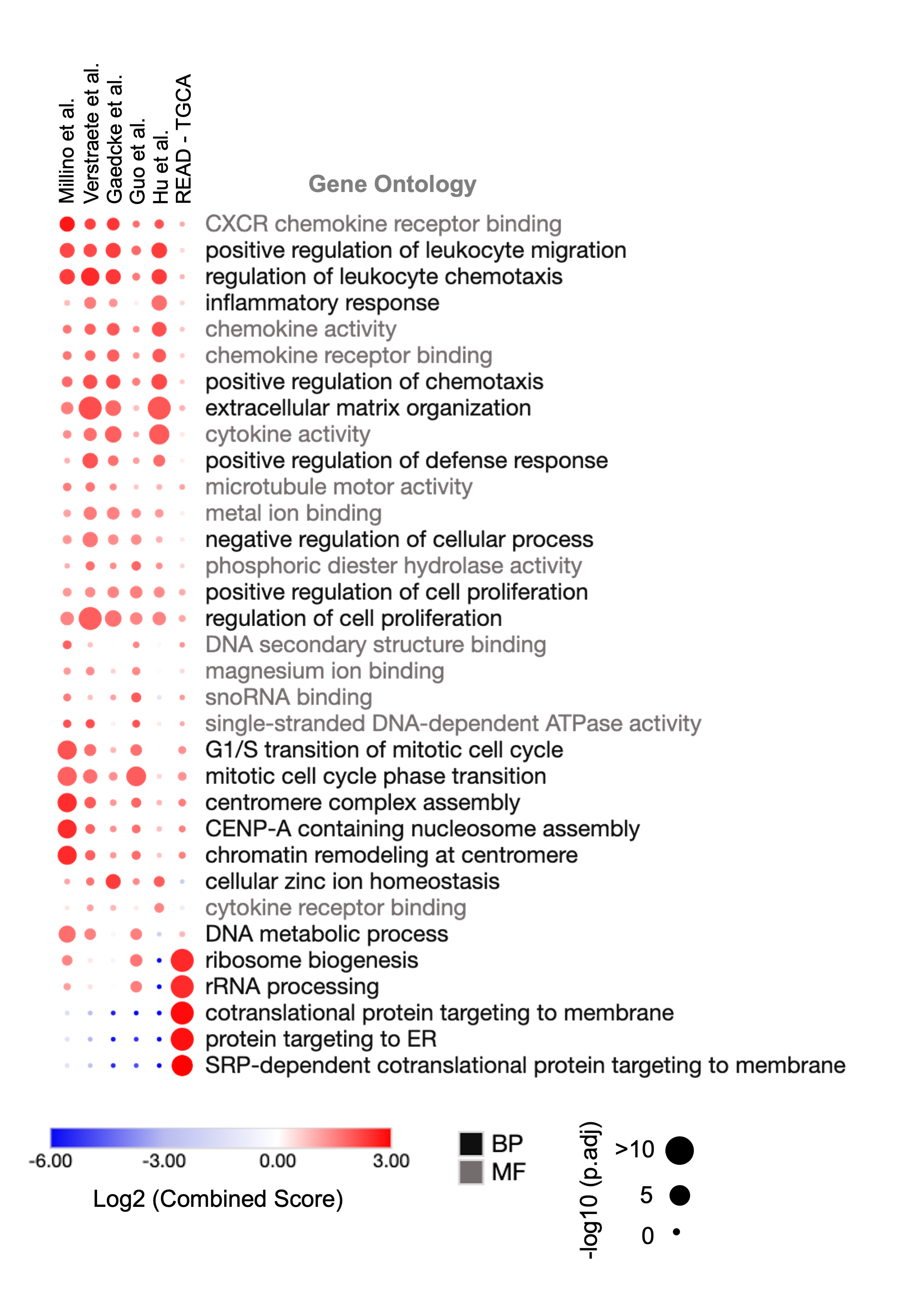

Supplement: Supplementary file 1 [file cancers-13-05492-s001.zip › Supplementary Figure S6.tiff]

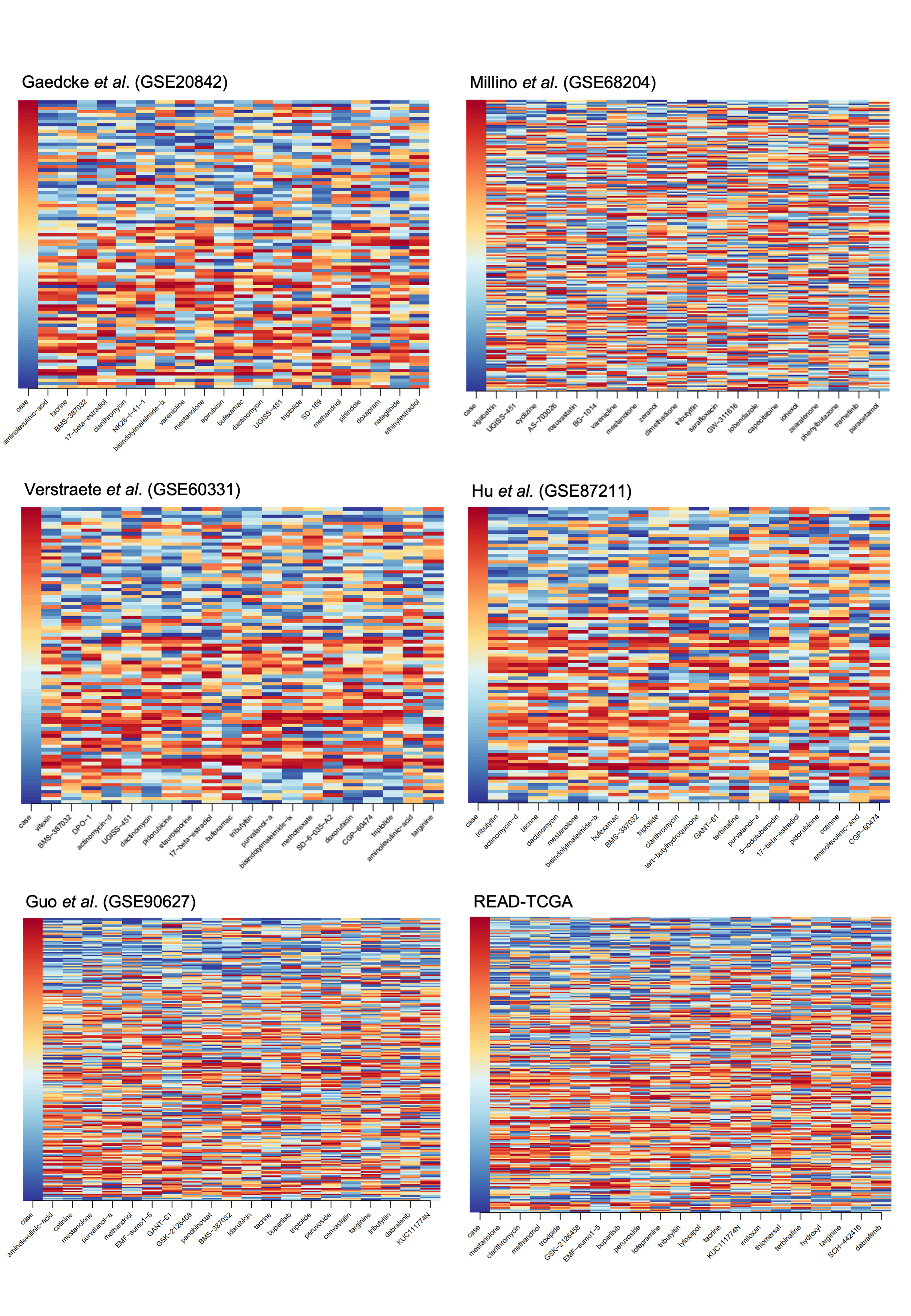

Supplement: Supplementary file 1 [file cancers-13-05492-s001.zip › Supplementary Figure S7.tiff]

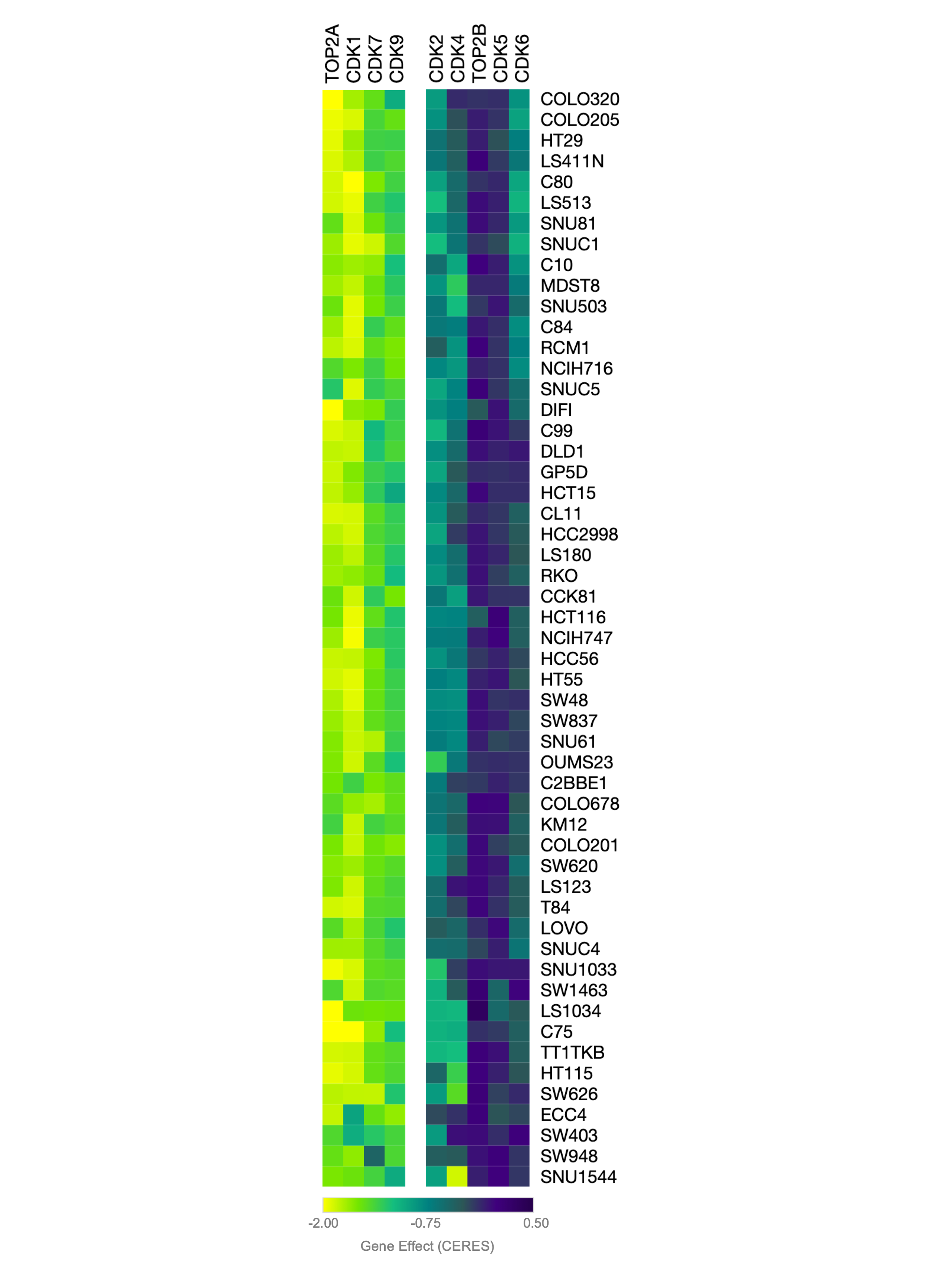

Supplement: Supplementary file 1 [file cancers-13-05492-s001.zip › Supplementary Figure S8.tiff]

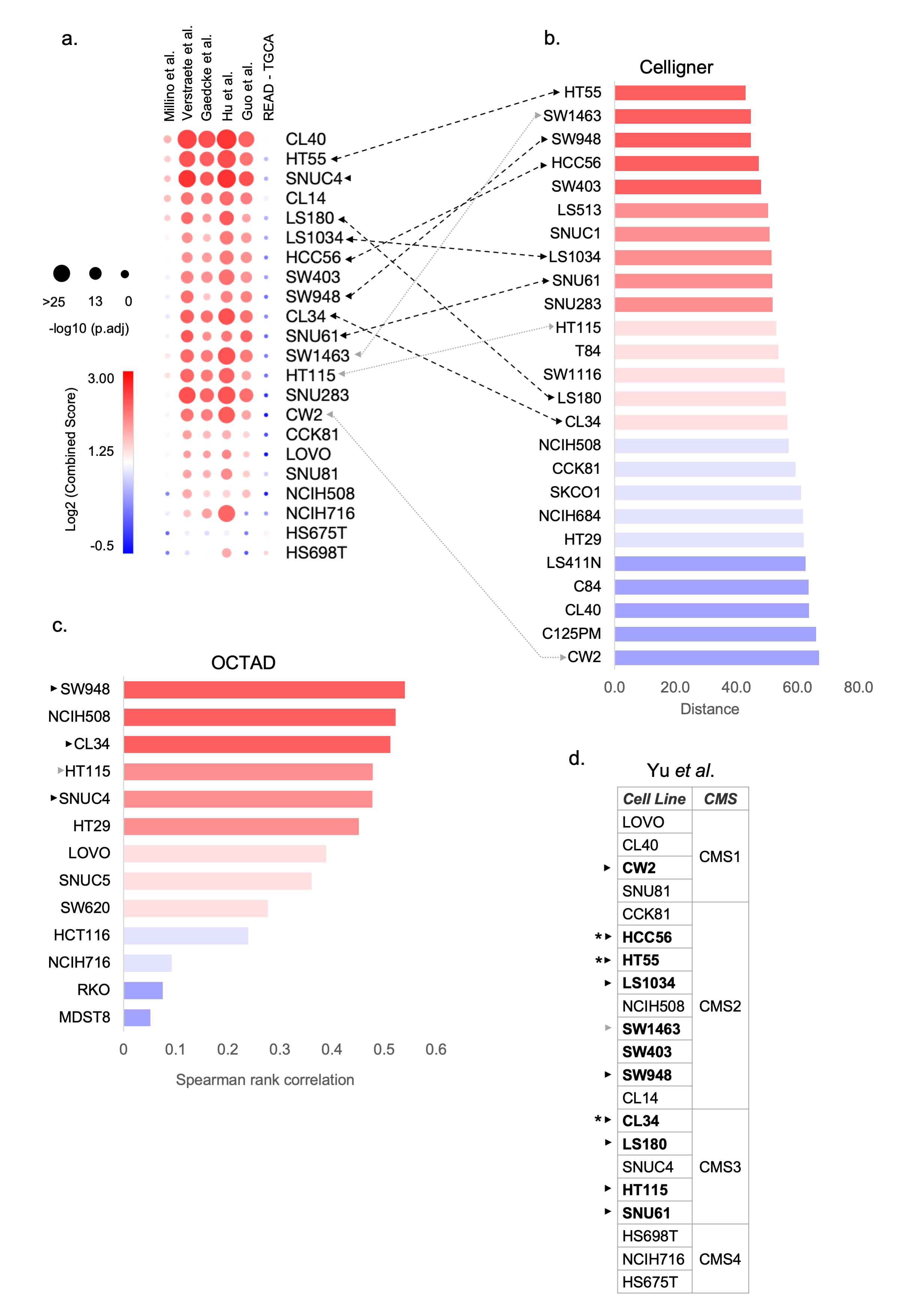

Supplement: Supplementary file 1 [file cancers-13-05492-s001.zip › Supplementary Figure S9.tiff]
